# Quantifying wood decomposition by insects and fungi using computed tomography scanning and machine learning

**DOI:** 10.1038/s41598-022-20377-3

**Published:** 2022-09-27

**Authors:** Sebastian Seibold, Jörg Müller, Sebastian Allner, Marian Willner, Petr Baldrian, Michael D. Ulyshen, Roland Brandl, Claus Bässler, Jonas Hagge, Oliver Mitesser

**Affiliations:** 1 Ecosystem Dynamics and Forest Management Group, Technical University of Munich, 85354 Freising, Germany; 2 Berchtesgaden National Park, Doktorberg 6, 83471 Berchtesgaden, Germany; 3 Terrestrial Ecology Research Group, Technical University of Munich, 85354 Freising, Germany; 4 Field Station Fabrikschleichach, Animal Ecology and Tropical Biology, Biocenter, University of Würzburg, Glashüttenstrasse 5, 96181 Rauhenebrach, Germany; 5 Bavarian Forest National Park, Freyungerstrasse 2, 94481 Grafenau, Germany; 6 MITOS GmbH, Lichtenbergstrasse 8, 85748 Garching, Germany; 7 Laboratory of Environmental Microbiology, Institute of Microbiology of the Czech Academy of Sciences, Videnska 1083, 14220 Praha 4, Czech Republic; 8 USDA Forest Service, Southern Research Station, Athens, GA USA; 9 Faculty of Biology, Department of Ecology, Animal Ecology, Philipps-Universität Marburg, Karl-Von-Frisch Strasse 8, 35032 Marburg, Germany; 10 Faculty of Biological Sciences, Institute for Ecology, Evolution and Diversity, Goethe University Frankfurt, 60438 Frankfurt am Main, Germany; 11 Forest Nature Conservation, Northwest German Forest Research Institute NW-FVA, 34346 Hann. Münden, Germany; 12 Forest Nature Conservation, Georg‐August‐University Göttingen, 37077 Göttingen, Germany

**Keywords:** Fungi, Biological techniques, Ecosystem ecology, Ecosystem services, Forest ecology, Entomology

## Abstract

Wood decomposition is a central process contributing to global carbon and nutrient cycling. Quantifying the role of the major biotic agents of wood decomposition, i.e. insects and fungi, is thus important for a better understanding of this process. Methods to quantify wood decomposition, such as dry mass loss, suffer from several shortcomings, such as destructive sampling or subsampling. We developed and tested a new approach based on computed tomography (CT) scanning and semi-automatic image analysis of logs from a field experiment with manipulated beetle communities. We quantified the volume of beetle tunnels in wood and bark and the relative wood volume showing signs of fungal decay and compared both measures to classic approaches. The volume of beetle tunnels was correlated with dry mass loss and clearly reflected the differences between beetle functional groups. Fungal decay was identified with high accuracy and strongly correlated with ergosterol content. Our data show that this is a powerful approach to quantify wood decomposition by insects and fungi. In contrast to other methods, it is non-destructive, covers entire deadwood objects and provides spatially explicit information opening a wide range of research options. For the development of general models, we urge researchers to publish training data.

## Introduction

In the world’s forests, 73 ± 6 Pg (Petagram, 10^15^ g) of carbon is currently stored in deadwood representing about 8% of the global forest carbon stock^1^. The decomposition of deadwood is, thus, a central ecosystem process determining release rates of carbon and nutrients^1–3^. Wood decomposition rates depend on climatic conditions^2^ and intra- and interspecific differences in wood traits (e.g. wood density, lignin, nitrogen or phosphorous content)^4–6^, but also on the communities of decomposers^2,7,8^. Microbes, particularly fungi, and arthropods, particularly termites and beetles, are the main biotic agents of wood decomposition^7,9,10^. While insects mainly degrade wood mechanically by creating larval tunnels and fragmenting woody material^7^, fungi degrade wood chemically, e.g. by use of extracellular enzymes or oxidative processes^10,11^. Moreover, interactions between organisms, for example insects and fungi or between fungi and bacteria, can affect wood decomposition^9,12,13^. Despite the importance of wood decomposition as an ecosystem process, many aspects regarding its drivers remain unclear.

Wood decomposition has been studied from a technical perspective focusing on construction timber^14^ as well as from an ecological perspective in laboratory experiments^13,15^. Ecological studies of wood decomposition and decomposer communities in the field, however, are logistically challenging compared to other substrates like leaf litter due to the size of deadwood items and the long time needed for the decay. In addition, the measurement of wood decomposition faces a number of methodological challenges^16–18^. The most widely applied method to quantify decomposition is estimating dry mass loss of an item or density loss over a certain time period^18,19^. Drying, however, alters decomposer communities and eradicates many species, especially when high temperatures are used^20^, making it a form of destructive sampling. Approaches based on dry mass loss thus do not allow to repeatedly assess the progress of decomposition within the same log as a whole and it is even problematic for initial measurements since endophytic fungi present in living wood influence fungal community assembly^21^.

**Figure 1:**
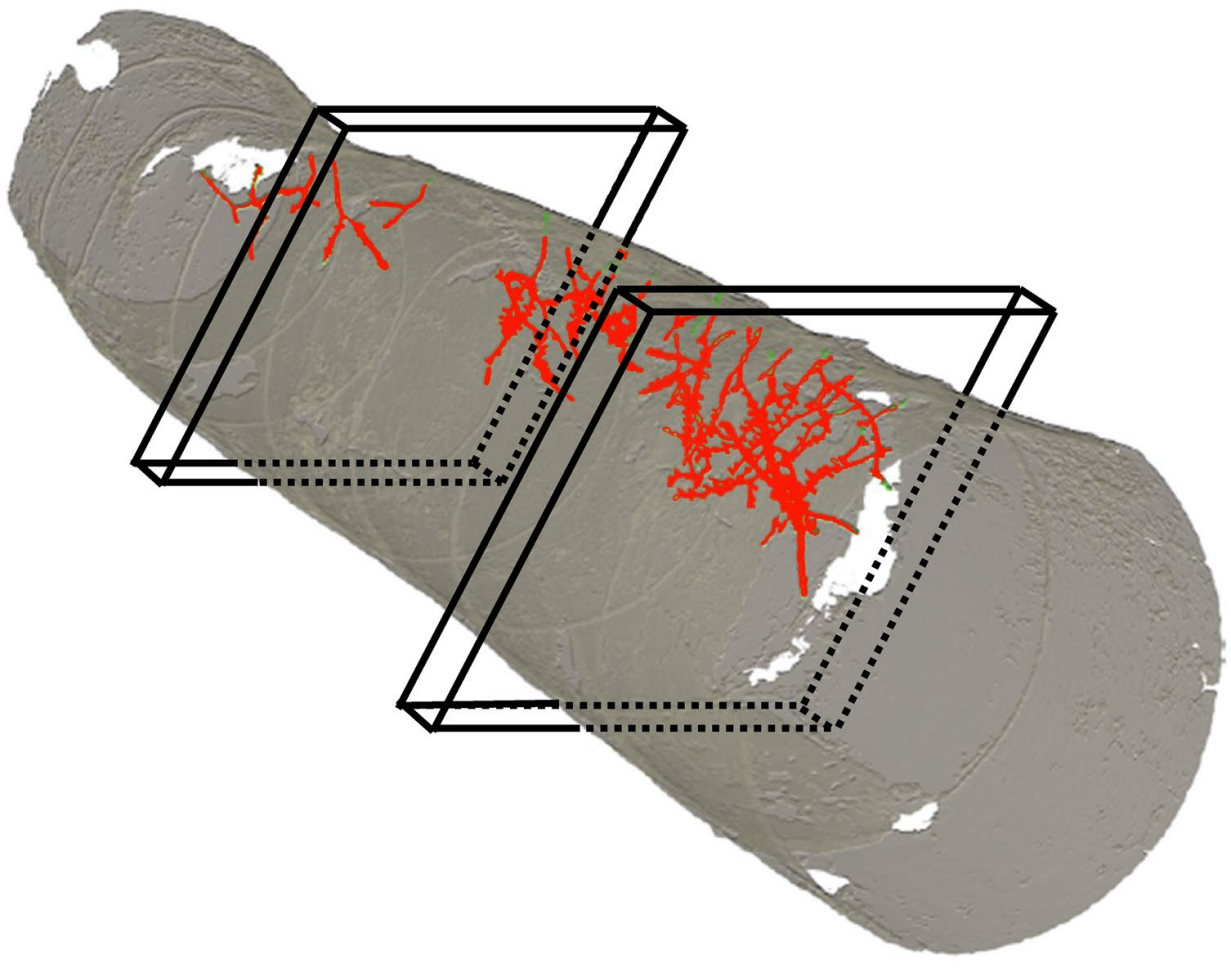
Heterogeneous distribution of tunnels generated by wood decomposing insects along the log can substantially affect the extrapolation of wood decomposition level from tree discs (indicated by black outlines). A specific disc might be located in areas with low or high decomposition activity. Scan by M. Gossner.

When decomposition under natural conditions or over several time steps is to be studied, subsamples have to be taken from a log, e.g. a disc or stem section, which are then dried to calculate dry mass or density of the study log based on the ratio of fresh and dry mass. Taking subsamples, however, suffers from limited accuracy since wood is a heterogenous substrate and heterogeneity within and between logs increases during the decomposition process^22,23^. Subsamples may thus not be representative of the rest of the log due to variation in wood characteristics, such as wood density, distribution of heart- and sapwood or presence/absence of branches. Especially when trying to quantify the decomposition by insects, this approach may lack accuracy since insects are not distributed homogeneously within wood^22^ (Fig. 1). There are non-destructive methods to measure wood density, such as resistograph, Pilodyn or nail-withdrawal measurements^24^, and also measuring chemical or biological characteristics of wood, such as pH, lignin, enzyme or ergosterol concentration^25–27^ is less destructive. Both types of approaches, however, suffer from limited representativeness since they are also based on subsampling, i.e. small amounts of wood extracted for example with a drill. Moreover, the measured chemical and biological characteristics mainly represent the activity of fungi and bacteria^26^, but not of insects. Another disadvantage of subsampling is that any mechanical disturbance of the bark can influence physical processes, such as drying, and the colonization of fungi by providing entry ports for spores^28,29^.

To provide a better understanding of the functional importance of different decomposer groups, it may be desired to quantify the contribution of each group separately. This is only partly possible with the currently available approaches to quantify wood decomposition. Approaches focusing on mass or density loss can only quantify total decomposition by all organisms. While exclusion of fungi in the field is unfeasible given the small size of fungal spores, insects exclusion, e.g. with cages, can be used to compare decomposition with and without insects^16^. The observed difference in decomposition rates with and without insects, however, is not only caused by the direct effects of insects but also by indirect effects mediated by insect-fungi interactions^2,7,30^. Past research attempting to elucidate patterns of wood decay and insect activity within logs involved making observations of fungal or insect damage visible on disks or boards cut from the logs^31–33^. In addition to being destructive, however, this painstaking approach is limited by the minimum thickness achievable using a saw and cannot be used for highly decomposed wood. Further progress in this area will thus require new methodology to quantify direct effects of insects and fungi.

In summary, approaches to quantify wood decomposition in a non-destructive way that represent complete deadwood items and are able to quantify both the results of fungal and insect activity are important to increase our understanding of the decomposition process. One promising approach is computed tomography (CT) scanning^19,34^, a method capable of measuring wood density or a proxy of wood density in a non-destructive way for logs up to several meters in length^35^. CT scanning is widely applied in the timber industry to measure wood quality, detect failures and optimize yield of sawn timber^36^. First attempts have applied CT scanning for deadwood, for example to monitor changes in wood structure due to decomposition by fungi^34^, to measure the extent and shape of termite nests^37,38^ or the temporal development of larval tunnels of the cerambycid *Monochamus scutellatus*^39^. Yet, ecological studies using CT scanning for quantifying wood decomposition of different decomposer groups are missing.

To evaluate the potential of CT scanning for quantifying wood decomposition by insects and fungi, we used experimental conifer deadwood placed in mesocosms to create defined beetle communities. Communities consisted of bark beetles or wood-boring beetles, as well as control logs without beetles, to create contrasts in the effect of insects on wood decomposition. We quantified for the first time wood decomposition by insects and fungi separately using CT scanning and semi-automatic picture recognition. To measure the volume of beetle tunnels, we applied a segmentation approach based on thresholding and to recognize areas affected by fungal activity, we used a deep learning approach (convolutional neural networks, CNN) which appears promising for assessing wood decomposition patterns^40^. To evaluate the potential of our novel approach, we compared the results of this approach to dry mass loss as a traditional measure for wood decomposition and to ergosterol measurements as a proxy for fungal activity.

## Results

**Figure 2:**
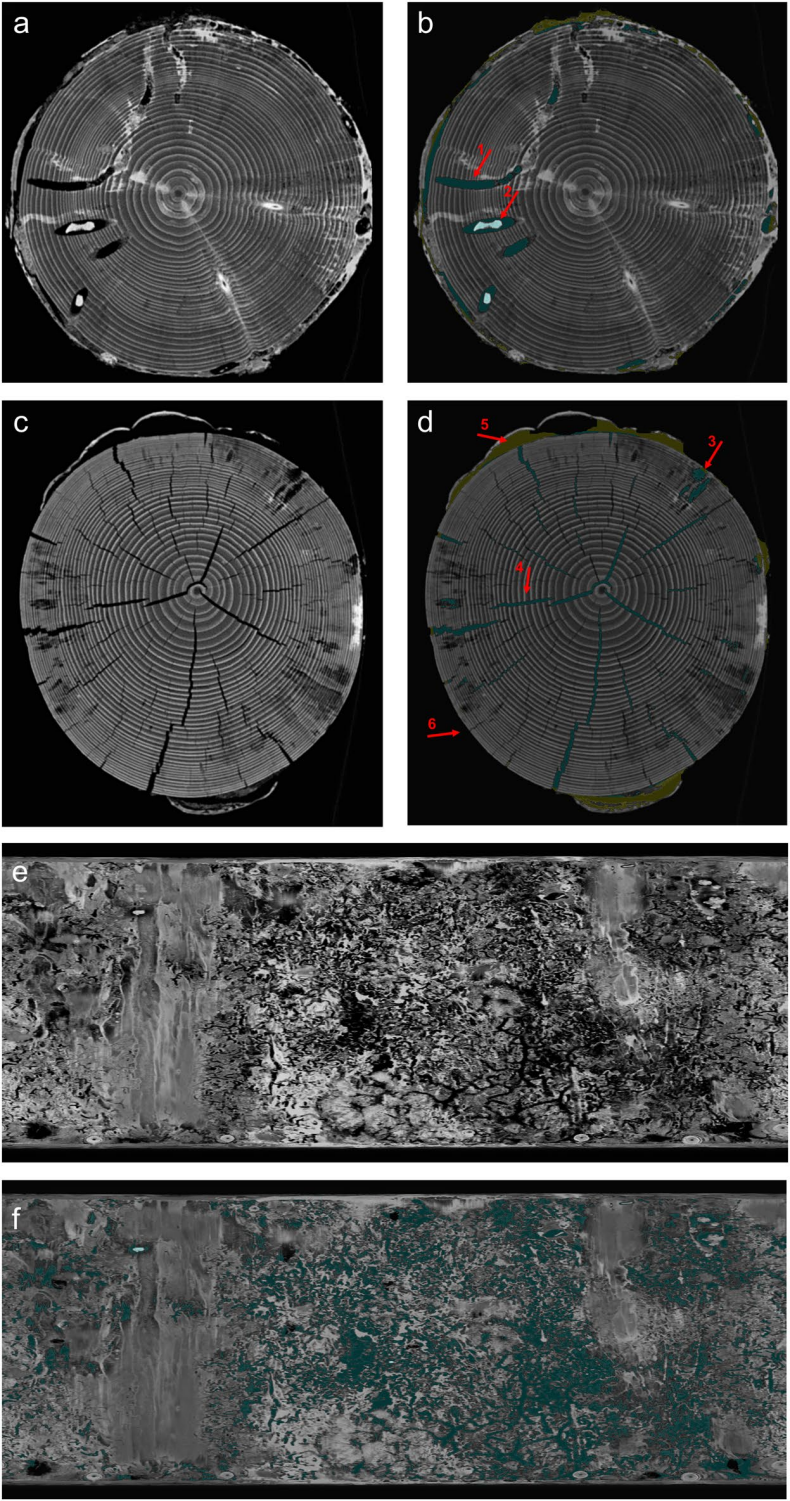
Computed tomography images of cross sections (**a**–**d**) and unrolled bark (**e**–**f**) after 1.5 years of decomposition. (**a**,**c**,**e**) Show raw images before segmentation and (**b**,**d**,**f**) show detected beetle tunnels in green and yellow. Correct identification is highlighted in (**b**) for beetle tunnels (1) and beetle larvae (2). Detection errors are highlighted in d including areas affected by fungal decay (3) and shrinkage cracks (4) falsely classified as beetle tunnels as well as challenges related to bark decay. Detached bark (5) can lead to overestimation of beetle tunnel volume under the bark while bark loss (6) leads to underestimation. (**e**,**f**) Show projections of the unrolled bark layer with the vertical axes representing the longitudinal stem axis.

**Figure 3:**
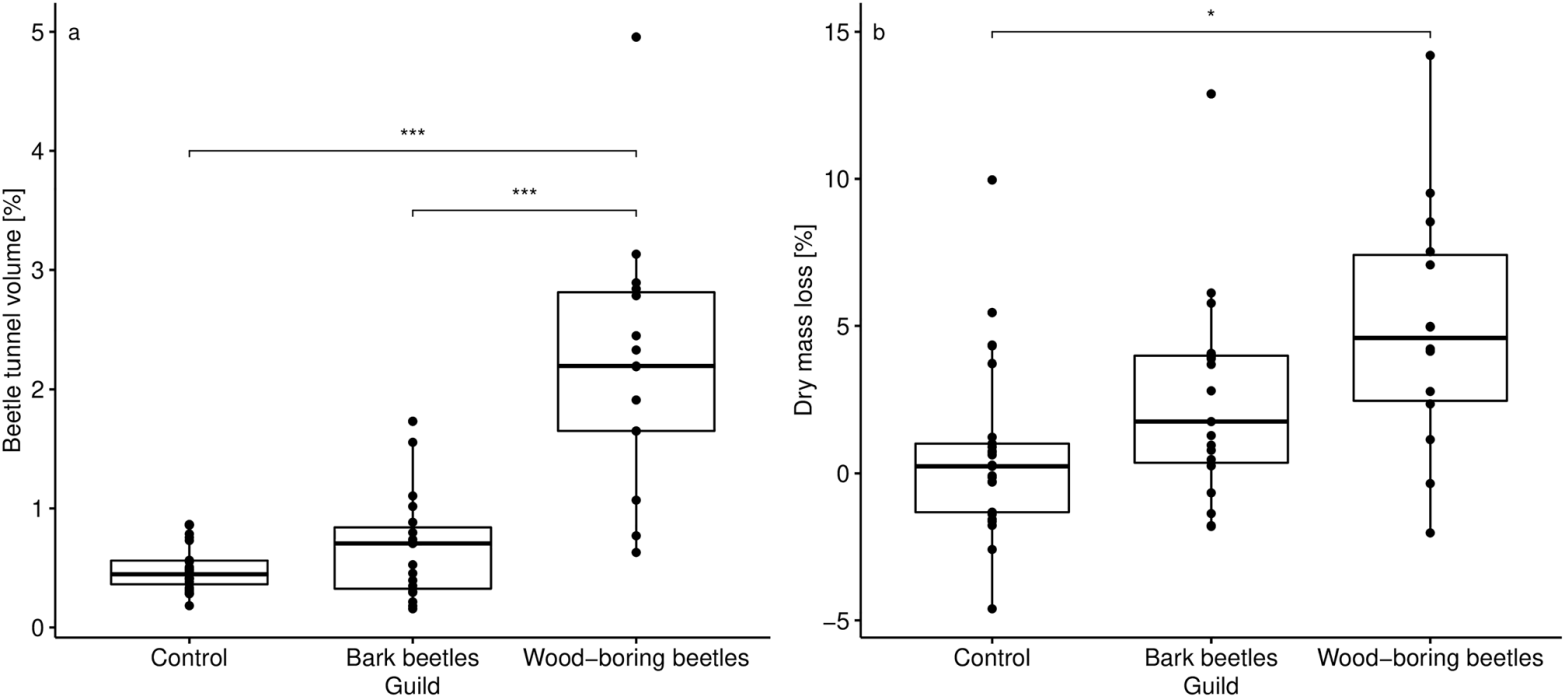
Relative volume of beetle tunnels [%] and dry mass loss [%] (both including bark) at the after 1.5 years of decomposition for mesocosms containing bark beetles or wood-boring beetles and the control without beetles. Significant results are indicated above the data groups.

For the logs of the 1st batch (after 1.5 years), decomposition by beetles was quantified by the volume of tunnels. Each pixel could be classified as either background, bark, wood, or insect generated tunnel in bark or wood (Fig. 2a,b,e,f). Misclassification due to shrinkage cracks and fungal decay, however, occurred (Fig. 2c,d) and are indicated by relative “beetle tunnel” volume of 0.5% in control logs without beetles (Fig. 3). In general, significant differences in beetle tunnel volume could be linked to beetle activity with differences between beetle guilds (Fig. 3). The fraction of tunnel volume was more than four times (and significantly) greater in mesocosms with wood-boring beetles compared to the control without beetles. Activity of bark beetles showed a tendency (not significant) with an increase in relative tunnel volume of about 50% compared to the control.

**Figure 4:**
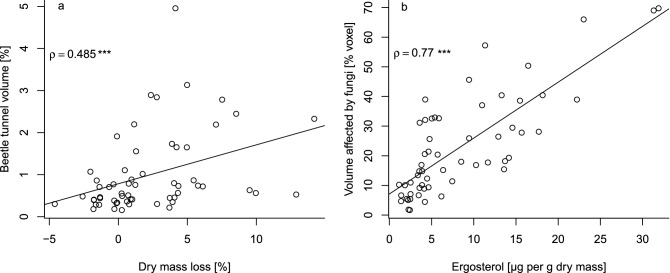
(**a**) Correlation between beetle tunnel volume and dry mass loss (both including bark). (**b**) Correlation between volume affected by fungi and ergosterol content. Correlations are based on Spearman’s rank tests and plotted lines are the regression line according to a linear model. Note that data in (**a**) were recorded after 1.5 years and data in (**b**) from different samples after 3.5 years.

**Figure 5:**
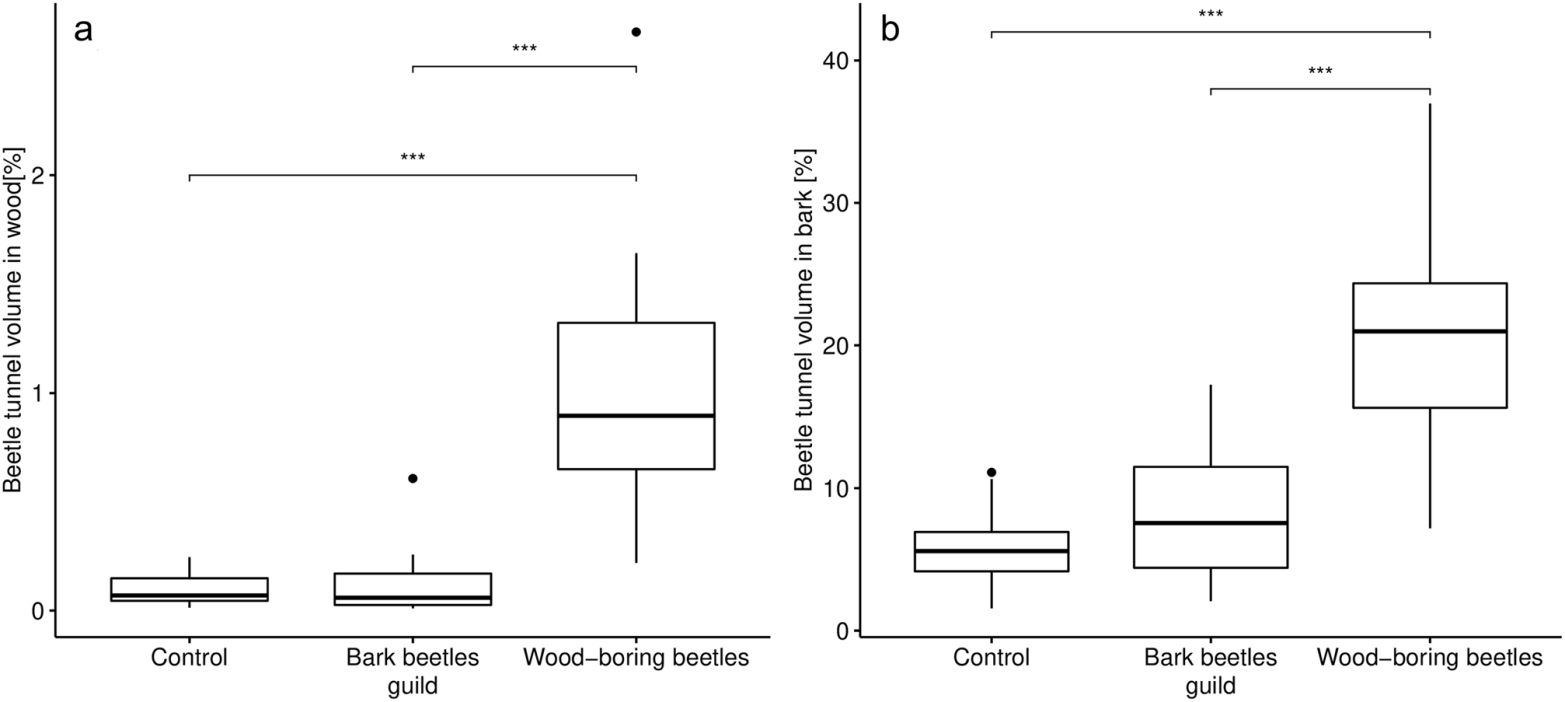
Relative beetle tunnel volume [%] after 1.5 years of decomposition for mesocosms containing bark beetles or wood-boring beetles and the control without beetles separately for bark and wood. P-values of pairwise and Holm-corrected *t* tests are given above the data groups.

The evaluation of dry mass loss in the control and both treatment groups revealed qualitatively similar results and identical significance rating (Fig. 3). Spearman rank correlation between beetle tunnel volume and mass loss was highly significant (p < 0.001) with coefficient ρ = 0.49 (Fig. 4). Separating the data set into wood vs. bark revealed significant differences between mesocosms with wood-boring beetles and the control for both wood (Fig. 5a) and bark (Fig. 5b). The differences between mesocosm with bark beetles and the control was higher when only bark was considered, but the effect was not significant (Fig. 5).

**Figure 6:**
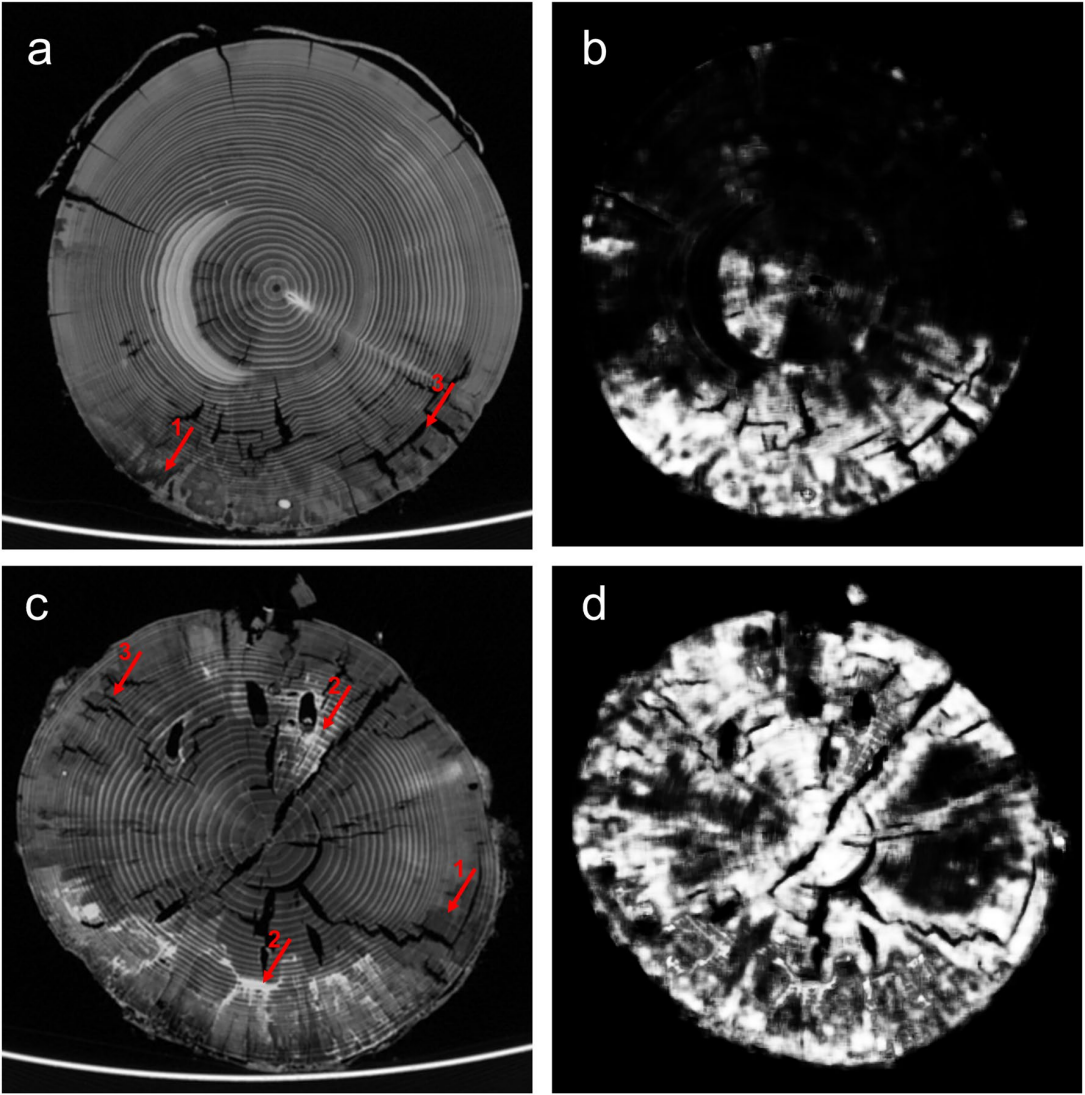
Computed tomography images of cross sections after 3.5 years of decomposition showing the raw images (**a**,**c**) and the corresponding prediction from the CNN model (**b**,**d**). White shading in b and d indicate a high probability of fungal decay, while dark shading indicate either wood with low probability of decay or cracks. Fungal decay was indicated either by darker (1) or brighter pixels (2) than the decayed wood or by perpendicular crack systems typical for brown rot (3). Note that cracks were not counted towards that area affected by fungal decay.

Decomposition by fungi after 3.5 years was characterized by pixels appearing either brighter or darker than the surrounding wood, indicating higher and lower density, respectively (Fig. 6; see also Appendix “Expert annotation”). Many logs showed patterns of cracks typical for brown rot fungi and we observed darker coloration for both early- and latewood. Average volume affected by decay was about 20% resembling the level in expert annotation. Evaluation of results of the CNN aiming to detect areas showing signs of decomposition by fungi using cross validation yielded an F1 score of 0.74 with accuracy of 93%, sensitivity of 77%, specificity of 95%, and precision of 70%. Correlation between the volume of wood showing signs of fungal decay and ergosterol content were highly significant (Fig. 4).

## Discussion

Methods to quantify wood decomposition by different decomposer groups in a non-destructive way that consider complete logs instead of subsamples have been lacking. Using a wood decomposition experiment with manipulated beetle communities, we tested the ability of CT scanning combined with semi-automatic image recognition to quantify wood decomposition by insects and fungi. Volumes of tunnels created by beetles derived from CT scanning data was significantly correlated to dry mass loss and clearly revealed that the relative importance of beetles to wood decomposition varies among functional guilds. The wood volume affected by fungal decay could be quantified utilizing a machine learning approach which yielded high classification accuracy and high correlation between estimated volume affected by fungal decay and ergosterol content (as a proxy for fungal biomass).

Dry mass loss is a traditional measure to quantify wood decomposition but with the disadvantage of being a form of destructive sampling due to the high temperatures killing decomposer communities and of being limited to rather small deadwood objects or subsamples of large objects^16,20^. Both beetle tunnel volume and dry mass loss revealed significant differences between control and wood-boring beetles, but only weak differences between control and bark beetles. The strong effect of wood-boring beetles compared to bark beetles reflects the ecology of both guilds with wood-boring beetles excavating larger amounts of wood and supports the hypothesis that large-bodied wood-boring beetles are the major decomposers among beetles^7^. Our results indicate that both approaches are in principle suitable to quantify wood decomposition by beetles as well as differences between guilds. While quantifying wood volume consumed by insects provides insight into the direct contributions of these organisms to wood loss from ecosystems, it should be noted that previous work suggests that insect-mediated reductions in wood volume do not guarantee losses in wood mass if fungal activity varies between treatments^32^. Our results, however, show that higher reductions in wood volume due to beetle activity were overall associated with higher mass loss, even if the strength of this correlation was only moderate.

For bark beetles, both approaches have their weaknesses. While neither approach found strong significant effects of bark beetles, dry mass loss revealed a slightly stronger effect than the CT-based approach. This may be attributable to losses of bark resulting from bark beetle activity^41^. While this may lead to overestimation of dry mass loss of wood and bark by bark beetles, it has mixed effects on our volume-based CT-approach. When bark is still attached but loose (Fig. 2d), the volume of bark beetle tunnels is overestimated. However, when the bark has fallen off before scanning, bark beetle tunnels cannot be quantified and thus effects of bark beetles are underestimated. Whether over- or underestimation prevails likely depends on the decomposition stage. Further improvement of post-processing of the image analysis may help to reconstruct or estimate the volume of missing bark.

A clear advantage of the CT-based approach is that beetle effects can be quantified for wood and bark separately, even if the accuracy is lower for bark. Wood-boring beetles contributed significantly to wood decomposition for both wood and bark. This mirrors the behavior of their larvae tunneling into the sapwood and in some species even into the heartwood, but also generating cavities under the bark, such as pupal chambers^42^. As expected, the effect of bark beetles was restricted to the bark although the observed trend was not significant. This may be due to challenges related to quantify the volume of cavities under the bark described above. Furthermore, not all bark beetle tunnels may be detected due to their small diameter when using CT scanners with limited spatial resolution. Similarly, visual inspection of images revealed that not all small-diameter tunnels were detected in wood. Advances of CT technology or the use of micro-CTs^34^ may be helpful to obtain a higher detection rate of small diameter tunnels.

Beetle tunnel volume larger than zero for control logs indicated that the thresholding approach produces false positives that were mainly associated with radial cracks due to shrinkage (Fig. 2d). Combining thresholding with geometry recognition that separates tunnels from cracks based on the shape may help to reduce this kind of error. Maximum dry mass loss for control logs in the 1st batch attributable to fungal decay were about 5% (see Fig. 3). We also observed negative dry mass loss, i.e. an increase in dry mass, which may to some degree be due to the input of biomass and external nutrients by colonizing fungi^43^. However, the more likely explanation is heterogeneity in wood density and water content within logs causing inaccurate dry mass values when using stem sections or other forms of subsampling to estimate dry mass of larger deadwood objects which is one weakness of the dry-mass based approach.

Visual inspection of images by experts revealed that fungal decay was associated with either brighter or darker pixels compared to unaffected wood (Fig. 6; Appendix “Expert Annotations”). Bright colors indicate high densities likely due to a high moisture content associated with recent fungal activity^34^. Darker pixels indicate lower densities which is likely a result of wood degradation by fungi. Density reduction was observed in either early- or latewood or in both. These different patterns could be caused by different fungal species but also the same fungus can cause different patterns with regard to early- and latewood depending on year ring width^34^. Using deep learning to quantify the volume of wood altered by fungal decay achieved reliable levels of accuracy and F1 score. Strong correlation between the relative volume of fungal decay and ergosterol content indicates that CT-based measures of fungal decay reflect fungal biomass in deadwood. The disadvantage of ergosterol content and other chemical or physical measures of fungal decay (e.g. enzyme profiles and activity) is that they are only based on subsamples of deadwood objects and thus do not provide information for complete deadwood objects^44^. Dry mass loss may represent complete logs (when complete logs are dried and weighed), but provides only an overall measure of decomposition combining all decomposer groups and information is not spatially explicit. Our approach combining CT scanning and deep learning provides volume-based and spatially explicit information on wood decay by fungi for complete deadwood objects. We showed that the accuracy of fungal decay recognition can be high, at least when training data of logs are used which are of similar size, age, decay stage and have a similar water content. The accuracy may be lower when samples are more heterogenous than in our case or when predictions are made outside of the environmental space defined by the training data. For example, water content associated with fungal activity depends on the decomposition stage and the involved wood-decomposing fungi which may thus affect CT images^34^. Training data should therefore be selected carefully to represent the full gradient of wood condition. To be able to develop general models that can be applied widely, training data including segmented and annotated images from different researchers should be made available in public databases. Note that a CT-based approach to quantify wood decay by fungi relies on visible changes in wood structure and may thus underestimate fungal activity during early stages of wood decay.

Binary segmentation of the CNN prediction can vary slightly for adjacent slices especially at the border between fungi-affected wood and unaffected wood. This happens because the continuous fungi prediction (confidence level) with a 2D CNN is slice-independent and binarized with a threshold (Fig. S2). This could be prevented by extending the network to 3D at the cost of increased labeling and training effort. A very similar effect could be reached by mildly blurring the network predictions along the longitudinal axis to even out irregularities. Therefore, this specifically seems appropriate as spread of fungi decay is more likely in longitudinal direction leading to less variation than is expected in radial direction^34,45,46^. As an alternative to classical segmentation and deep learning, classification by traditional machine learning, e.g. a random forest classifier, could be used. As a supervised learning approach, it utilizes generic (nonlinear) features and provides good accuracy based on ground truth labels with little training data. One example for a machine learning classifier with an interactive training editor with live feedback is Ilastik^47^. However, we selected a deep learning approach as it bears the potential to outperform this approach given a sufficient amount of training data.

**Figure 7:**
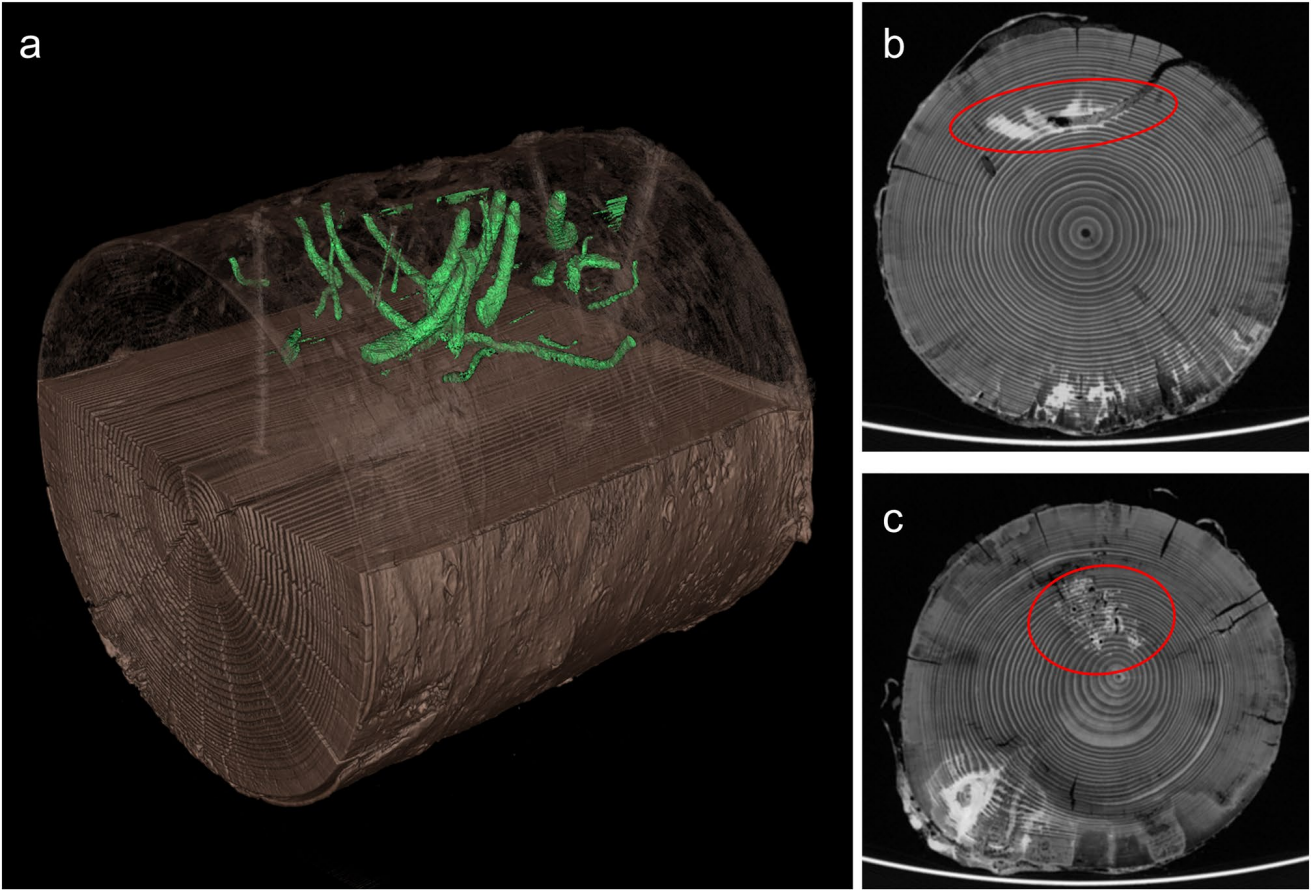
Computed tomography allows spatial analyses of beetle and fungal activity patterns and species interactions. (**a**) Shows the 3-D structure of beetle tunnels in a deadwood log reconstructed from automatically detected tunnel structures (generated with X-AID 3D volume viewer). In (**b**) and (**c**), areas of fungal decay (white) are located close to beetle tunnels indicating that both taxa are closely interacting.

The spatially explicit information where fungi and insects are active in deadwood opens a wide range of options for further research. This includes the analysis of ecological differences between species, e.g. differences in tunnel sizes, which may explain the functional role as well as the interactions between insect species and between insects and fungi. Three-dimensional patterns of tunnels of different beetle species (Fig. 7a) may show whether different insect species avoid each other, for example due to competition or intra-guild predation^48^. Moreover, linking spatial information of insect tunnels to patterns of fungal communities and decay (Fig. 7b,c) can help to understand the role of insects as vectors and insect tunnels as entry ports for fungal spores and hyphae^28,31^. Another field of research includes spatial patterns of decay within deadwood and its drivers^34^. Fungal and insect species differ in moisture and temperature preferences and thus, colonize different parts of deadwood logs^42,49^. Linking spatial distribution patterns of insects and fungi to microclimatic conditions within deadwood can help to better understand how habitat heterogeneity drives biodiversity. Finally, sampling of fungal communities and chemical variables could be spatially informed, i.e. sampling could be conducted at locations that show low and high fungal activity.

## Conclusion

We tested a novel approach combing CT scanning with semi-automatic image analysis as a measure to quantify the role of beetles and fungi in the process of wood decomposition. Our measures of beetle tunnel volume and wood volume affected by fungal decay revealed high accuracy and strong correlation with traditional measures (i.e., dry mass loss and ergosterol content, respectively). Moreover, differences between functional guilds of beetles were captured. Compared to existing methods, this approach has several advantages: (i) it is possible to quantify the contribution of insects and fungi to wood decomposition separately; (ii) sampling is non-destructive and can thus be used to generate time series without interrupting succession and altering conditions; (iii) it is not based on subsampling (i.e. stem sections), but represents entire logs and is thus unbiased by heterogeneity within deadwood objects; (iv) it provides spatially explicit information which opens plenty of options for further studies (e.g., regarding interactions between species or heterogeneity within deadwood). Despite the many advantages offered by this approach, its utility is likely to diminish as decomposition proceeds and it becomes increasingly difficult to distinguish between insect galleries and cracks in the wood. Currently, the size and the fragility of highly decomposed bark and wood are logistically challenging as long as logs have to be transported to be scanned by a stationary scanner. Mobile CT-scanners, however, are currently developed which may soon allow to scan logs, even of large size, in the field. To make use of future progress in CT-technology and automatic image recognition, we urge researchers to share segmented imagery as training data for developing general models for detecting decay by insects and fungi which can be applied for a wide range of substrates.

## Methods

### Decomposition experiment

To achieve controlled conditions of wood decomposition, we set up an experiment using mesocosms in the Bavarian Forest National Park, in southeastern Germany. In March 2015, foresters of the national park felled 60 to 80 years old Norway spruce (*Picea abies*) trees without signs of insect or fungal infection at a single forest stand. From these trees, we cut 122 logs of 53 cm length ranging from 16 to 20 cm in diameter. We cut a 3 cm long disc at the edge of each log. The fresh mass was recorded for each disc and each remaining 50 cm log with accuracy of 0.1 g. Discs (including bark) were dried at 65 °C until mass remained constant and dry mass was measured. We calculated the dry mass of the respective logs (including bark) as *dry mass log* = (*fresh mass log*/*fresh mass disc*) × *dry mass disc*. To control for colonization of logs by insects, we placed logs inside cages (mesocosms) made up of white polyester mesh with 1000 mesh per square inch measuring 40 cm × 40 cm × 60 cm^2^. Two logs were assigned randomly to one of 61 mesocosms. 26 mesocosms served as control without beetles, 20 mesocosms were assigned to the treatment *bark beetles* and 15 mesocosms to the treatment *wood-boring beetles.* All mesocosms of the treatment *bark beetles* contained 20 individuals of *Ips typographus* and of none to three further bark beetle species: *Hylastes cunicularius* (10 cages, 10 individuals each), *Dryocoetes* spec. (7 cages, 10 individuals each), *Pityogenes chalcographus* (15 cages, 20 individuals each). All mesocosms of the treatment *wood-boring beetles* contained four individuals of *Monochamus sutor* and five individuals of none to seven further wood-boring beetle species: *Tetropium castaneum* (10 cages), *Rhagium inquisitor* (10 cages), *Molorchus minor* (5 cages), *Acanthocinus griseus* (2 cages), *Clytus lama* (4 cages), *Antaxia quadripunctata* (10 cages), *Chrysobothris chrysostigma* (1 cage), *Callidium violaceum* (4 cages). These species were chosen because they are typical of the early-successional community colonizing dying conifers as well as fresh coniferous deadwood and they are feeding on the phloem, cambium or xylem (Table S1). Beetles were collected in the same study region using pheromone traps for Scolytinae and hand collection for Cerambycidae and Buprestidae. The experiment was conducted at five plots within the Bavarian Forest National Park with 5, 4 and 3 replicates per plot of control, bark beetle and wood-boring beetle treatments (one plot received 6 mesocosms of the control treatment), respectively.

In November 2016, i.e. 1.5 years after colonization of wood by beetles, we collected one log from each mesocosm (“1st batch”). Logs were carefully wrapped in paper to avoid the loss of bark or frass during transport. Logs were then scanned individually by a Philips iCT SP computed tomography scanner with helix scan mode 120 kVp, slice thickness of 0.67 mm and pixel spacing of 0.29 mm. After scanning, logs were dried at 65 °C until mass remained constant at Technische Hochschule Rosenheim and dry mass was determined. Note that one advantage of a CT-based approach to quantify wood decomposition is that individual logs can be monitored over longer periods since it is a non-destructive form of sampling. Here, we scanned each log only once since we aimed at comparing the CT-based approach to dry mass loss which required drying the logs and thus interrupting the decomposition process.

The second log from each mesocosm was collected in November 2018, i.e. 3.5 years after the wood was colonized by beetles (“2nd batch”), and scanned by a Philips Ingenuity Flex X-ray computed tomography scanner with helix scan mode 120 kVp, slice thickness of 0.8 mm and pixel spacing of 0.34 mm. As an estimation of variation in fungal biomass^50^, ergosterol concentration was measured for two wood samples of each log. Wood samples were taken before scanning by drilling 15 cm and 35 cm from one end of each log using a 8 mm auger operating perpendicular to the stem axis. Both samples of a log were pooled and total ergosterol was extracted with 10% KOH in methanol and analysed by high‐performance liquid chromatography (HPLC)^51^.

All methods were carried out in accordance with relevant guidelines and regulations.

### Processing of CT-data

Each pixel of the reconstructed CT images (1024 × 1024 pixels) indicated the opacity level of the material in Hounsfield Units (HU), which are scaled relative to water attenuation level. In a first step of the analysis of both batches, the bearing area (i.e. the patient table) of the CT device was identified by a characteristic opacity level resulting from the synthetic surface after averaging over all slices. The area below (and including) the borderline was replaced by the transmission level of the surrounding air (black). Beetle species included in the experiment are most active during the first two years of succession^52^, while decomposition by fungi increases with succession^53^. Thus, processing of CT data obtained in 2016 (1st batch) focused on detecting beetle tunnels and quantifying tunnel volume, while processing of CT data obtained in 2018 (2nd batch) focused on recognizing and quantifying wood affected by fungal activity.

#### Approach 1: Recognition of beetle tunnel volume (1st batch)

Tunnels created by beetles in the 1st batch were identified by a segmentation approach which is typically applied for detection problems with high-contrast images and can be implemented rapidly with reproducible outcome. Thresholding in combination with binary morphological operations like erosion, dilation, opening, or closing were utilized to create feature maps^54^ distinguishing between pixels with and without a specific desired attribute, starting with wood detection and separation of bark from inner wood, for details see Appendix “Wood and bark detection”. Air filled cavities appeared as dark gray pixels and could thus be detected by a similar hysteresis thresholding approach as for wood detection^54^, see Appendix “Cavity detection”. After cavity detection, drillings and shrinkage cracks could be identified by their geometrical characteristics and thus be separated from beetle tunnels. Remaining cavities were tagged as beetle tunnels. However, larvae within tunnels as well as refilled tunnels were not discriminated from wood yet. Larvae provide good contrast and appear bright in the CT images. A larva mask was generated by thresholding and refined by checking for direct connection to a detected tunnel and validation of size. Thus, erroneous classification of other moisture-containing parts of the wood as larvae could be avoided. Refilled tunnels could not be detected robustly; however, they contributed negligibly to the entire cavity volume and could be neglected. Finally tunnel volume was determined by counting pixels representing beetle tunnels separately for the bark, wood and the complete log. In addition, a graphical three-dimensional representation of beetle tunnels could be generated by combining 2D mask images utilizing the 3D volume viewer from MITOS GmbH, Garching Germany. Virtual bark unrolling (Fig. 2e,f) provided a radial view on the log.

#### Approach 2: Recognition of wood affected by fungal decay (2nd batch)

In 2018, the decomposition process had obviously advanced with the physical structure of the wood often being strongly deteriorated. Many logs showed typical signs of brown rot, such as rectangular crack systems and dark brownish color. Processing of CT data thus aimed at detecting regions affected by fungal decay and quantifying the corresponding volume. Classical segmentation did not reveal solid results for the 2nd batch since cracks and beetle tunnels strongly intermingled (results not shown). Most challenging in segmentation of the 2nd batch was the detection of distributed areas affected by fungal decay which was represented by a diverse range of gray levels and textures. For binary segmentation of visible fungal decay present vs. absent, we chose a deep learning approach which has been shown to recognize complex structures more reliably^55^.

Before feeding image segments into the machine learning algorithm they were offset corrected and normalized by a factor of 2500 HU to yield values in a range from 0 to 1. The upper interval boundary of 1 corresponds to a maximum level of 250% of water attenuation and provided the upper limit of a sufficient opacity range. Dark values near 0 indicated air. We labeled 197 cross-sections of the 2nd batch to cover a wide feature range by providing pixel-wise expert annotations (see Appendix “Expert annotation”). Each pixel was either labeled as background or fungal decay. A 2D convolutional neural network (CNN) was applied to operate on 48 pixel × 48 pixel input patches providing a sufficient amount of expert labeled training data. The adopted CNN architecture was very similar to the U-Net architecture^55^ and is illustrated in Appendix “Convolutional neural network” (Fig. S1). The output of the neural network indicated the probability of fungal decay being present in each pixel of the image. The threshold value for transforming probability values (interval [0, 1]) to a binary segmentation decision was chosen to maximize the F1 score of the validation dataset^56^. The F1 score is the harmonic mean of precision, i.e. the fraction of pixels classified correctly as affected by fungi among the pixels classified as affected by fungi, and sensitivity, i.e. the fraction of pixels classified correctly as affected by fungi among all pixels actually affected by fungi. In addition, we evaluated accuracy (proportion of correct predictions among the total number of cases, i.e. fraction of pixels representing true fungal affection correctly classified among all pixels actually affected by fungi) and specificity, i.e. fraction of those that were not judged correctly as not affected out of those that are actually not affected (true negatives). Quality indicators are formally defined in the Appendix. Prediction reliability slightly decreased towards the borders of the image patches. Therefore, the 48 × 48 patches were extracted in an overlapping fashion with an offset of 12 pixels between corresponding pixels of two neighboring patches in a row or column. This ‘shift’ by 12 pixels results in an overlap of 75% of the pixels in the two patches. Predictions were recombined after distance-from-center-driven cosine weighting of contributing pixels. This increased prediction time but substantially improved image quality. Finally, the volume of wood showing signs of fungal decay could be estimated by counting those pixels that indicated the presence of fungal decay.

Image processing was realized with Python 3.6^57^. For basic image manipulation (e.g. morphological operations like opening or closing) we used the implementation of the scientific Python package Scipy (version 1.3.1^58^). Deep learning package Keras (version 2.2.5^59^) was utilized with machine learning platform TensorFlow^60^ to implement the convolutional neural network.

### Statistical analyses

Statistical analyses were conducted in R version 3.6.3^61^. To compare the relative volume of beetle tunnels identified by segmentation for bark, wood, and the entire log between beetle guilds and control treatment we utilized a beta regression model, function *gam* with family *betar* in package *mgcv* (version 1.8-31) to account for proportional data and Tukey post-hoc test for pairwise comparisons. The same test was applied to relative dry mass loss. Correlations between relative beetle tunnel volume and dry mass, as well as between ergosterol and relative fungal decay volume were evaluated by Spearman rank tests.

## Supplementary Information

Supplementary Information.

## Data Availability

The entire labelled data set for training and testing the CNN is compiled here: https://figshare.com/s/d413d84da8476eaaef5e. All other datasets used during the current study are available from the corresponding author on reasonable request.
