# Supplementary material for: Quantifying wood decomposition by insects and fungi using computed tomography scanning and machine learning
Source: Sci Rep. 2022 Sep 27;12:16150. doi: 10.1038/s41598-022-20377-3 (PMC9515192; doi:10.1038/s41598-022-20377-3)
Supplement: Supplementary file 1 — Supplementary Information. [file 41598_2022_20377_MOESM1_ESM.docx]

**Supplementary Material**

**Quantifying wood decomposition by insects and fungi using computed tomography scanning and machine learning**

Sebastian Seibold, Jörg Müller, Sebastian Allner, Marian Willner, Petr Baldrian, Michael Ulyshen, Roland Brandl, Claus Bässler, Jonas Hagge, Oliver Mitesser

Corresponding author: sebastian.seibold@tum.de

**Beetle species**

**Table S1:** List of bark and wood-boring beetle species used in the experiment with information larval development time, used wood stratum (bark, bark-wood ecotone, sapwood or heartwood) and mean body size ^1–3^.

| Species | Family | Functional group | larval development time [years] | stratum bark | stratum bark-wood ecotone | stratum sapwood | stratum heartwood | body size [mm] | No. of individuals per mesocosm |
| --- | --- | --- | --- | --- | --- | --- | --- | --- | --- |
| *Monochamus sutor* | Cerambycidae | wood borer | 2.5 | 0 | 1 | 1 | 1 | 19.5 | 4 |
| *Tetropium castaneum* | Cerambycidae | wood borer | 1 | 0 | 1 | 1 | 0 | 13.5 | 5 |
| *Callidium violaceum* | Cerambycidae | wood borer | 2 | 0 | 1 | 1 | 0 | 12 | 5 |
| *Rhagium inquisitor* | Cerambycidae | wood borer | 2 | 0 | 1 | 1 | 0 | 15.5 | 5 |
| *Chrysobothris chrysostigma* | Buprestidae | wood borer | 2 | 0 | 1 | 1 | 0 | 12.75 | 5 |
| *Clytus lama* | Cerambycidae | wood borer | 2 | 0 | 1 | 1 | 0 | 11 | 5 |
| *Acanthocinus griseus* | Cerambycidae | wood borer | 1.5 | 0 | 1 | 1 | 0 | 10.5 | 5 |
| *Molorchus minor* | Cerambycidae | wood borer | 2 | 0 | 1 | 1 | 0 | 11 | 5 |
| *Anthaxia quadripunctata* | Buprestidae | wood borer | 1 | 0 | 1 | 1 | 0 | 6.25 | 5 |
| *Ips typographus* | Scolytinae | bark beetle | 1 | 1 | 0 | 0 | 0 | 4.85 | 20 |
| *Hylastes cunicularius* | Scolytinae | bark beetle | 1 | 1 | 0 | 0 | 0 | 3.85 | 10 |
| *Dryocoestes spec.* | Scolytinae | bark beetle | 1 | 1 | 1 | 0 | 0 | 3.5 | 10 |
| *Pityogenes chalcographus* | Scolytinae | bark beetle | 1 | 1 | 1 | 0 | 0 | 1.66 | 20 |

**Wood and bark detection in 1^st^ and 2^nd^ batch**

Wood, i.e. the log area, within an image was discriminated from the surrounding air by a multi-step process. Straightforward thresholding of the wooden material yielded a rough binary mask characterizing each pixel as wood (including bark) or non-wood. In order to remove small unconnected parts (e.g. fallen-off bark pieces or remainder of the CT-scanner bed) a binary opening was performed on the retrieved rough mask to gain a map of central wooden parts. A hysteresis* threshold ^4^ yielded a mask of central wood only separating it from the bark, however still containing cavities either from bug burrows, shrinkage cracks, or wood extraction drillings. Final binary closing with large kernel size revealed a central wood disk without exterior tails or holes eventually accompanied by bigger bark parts next to the log. Detection was finalized by cropping the image to a rectangle with distance of 30 pixels between log boundary and edges of the image typically resulting in square images with side length of 600 pixels.

**Cavity detection**

An aggressive wood threshold was used to detect even small decay in intensity and relatively narrow tunnels. However, without refinement this would have included small regions of darker parts of annual growth rings or image noise. Therefore, a core-feature mask with an increased gray level threshold was applied to qualify connected areas as cavities and exclude areas not containing dark gray subareas. This approach identified shrinkage cracks, wood-extraction drillings and bug burrows as cavities. Unfortunately, it missed smaller bug burrows with a hole diameter of less than two to three pixels (c. 1 mm). However, tuning the thresholding to include these small features would have caused many false positive cases as identified by visual inspection.

**Drilling and shrinkage detection**

Large and straight drilling voids met the wood center at a right angle. The number of this type of voids was known exactly. Thus, a drilling could be identified by region size and a orientation deviating by only a few degrees of inclination with respect to the slice axis in the log. Four out of 190 cases had to be revised manually due to deviations from the expected number of drillings. Shrinkage cracks had a specific orientation along the axial direction of the log (i.e. growth direction of the tree) which could be exploited to separate them from differently oriented features. Smoothing in axial direction smeared out other cavities, but did not change crack intensities. After smoothing simple thresholding revealed larger cracks.

**Virtual bark unrolling**

For virtual bark unrolling and a radial view, identification of the position of the central annual growth ring of each slice is crucial. However, a precise fully automated algorithm is hard to implement and complicated to verify. We followed a semi-automated approach, determined the position of the center for a certain number of slices per log manually, and interpolated to other slices by spline regression. This procedure was a good compromise between manual work and implementation effort, as it only needed a few seconds of expert interaction time per log and yielded reliable results.

With reference to the identified central positions, the dataset was resampled slice by slice in a radial way by angular sampling. The number of different angles was determined by the distance of tangential sampling points in the most outer regions of the logs to ensure that they were not further apart than pixels in the axial slice. A reasonable number of sampling angles was 2000 corresponding to logs with an approximate diameter of 600 pixels.

**Training data for convolutional neural network**

To generate training data for the convolutional neural network detecting areas affected by fungal decay, 197 images from different logs were visually inspected for signs of fungal decay and respective areas were marked. Two different forms of fungal patterns were classified separately: in the first, the pixels appear brighter than the remaining wood while in the second, the pixels appear darker then the surrounding. The brighter areas likely have a higher water content and thus high density, while the dark areas have a low density as a result of the decomposition process ^5^. Areas were considered affected by fungi according to the criteria described below.

**Table S2:** Criteria for classifying areas as “affected by fungal decay” during the expert annotation process.

| **Bright fungal pattern** | **Examples** |
| --- | --- |
| Overall appearance white |  |
| Early wood as bright as late wood OR late wood even brighter as usually  🡪 year ring structure disappears or is invisible  🡪 shape areal or undulating | 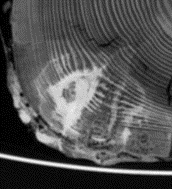 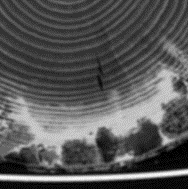  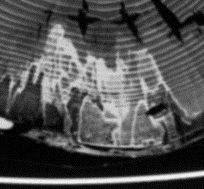 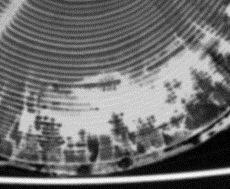 |

| **Dark fungal pattern** | **Examples** |
| --- | --- |
| Overall appearance dark |  |
| Late wood as dark as early wood 🡪 year ring structure disappears | 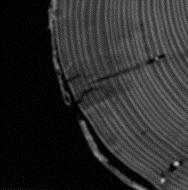 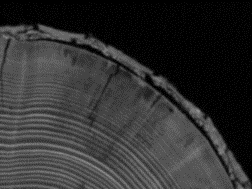 |
| OR early wood darker than usual 🡪 year ring contrast is stronger | 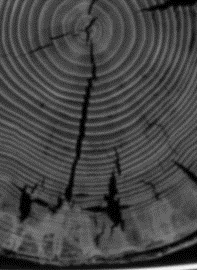 |
| OR early and late wood darker than usual | 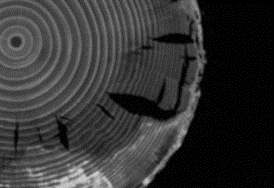 |
| OR grainy structure due to heterogenous distribution of draker pixels | 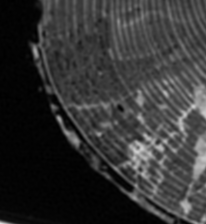 |
| Shape: triangular („piece of cake”) | 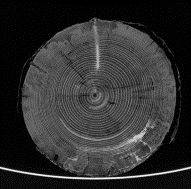 |
| Shape: OR system of rectangular cracks typical for brown rot | 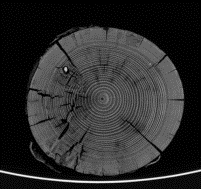 |

Cracks wider than 4 pixels were not excluded from the area classified as “dark fungal pattern”. In addition to the area affected by fungi, we classified areas which were not affected by fungi but resembled patterns typical for fungi, such as branches appearing white. To avoid that rare non-fungi areas are underrepresented, explicit non-fungi areas were classified but not included in the analyses.

**Convolutional neural network**


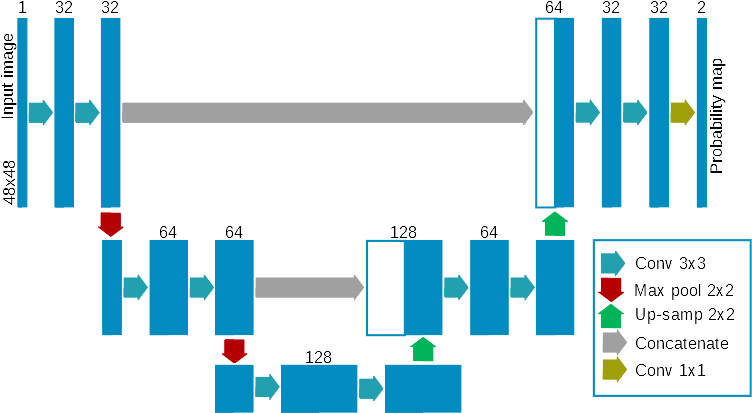


**Figure S1:** A U-net with depth of three levels. The input was a sequence of image fragments of 48x48 pixels extracted from the recorded CT images. In the three contributing levels, an image was processed with filter depths of 32, 64, and 128. The 3x3 convolution was followed by a ReLU (Rectified Linear Unit) evaluation and max pooling reduced the image size by selecting a maximum of 2x2 pixels. The up-sampling reversed the operation, creating 2x2 pixels from a single pixel of the lower level of the U-net. The final convolution recombined the 32 filters of the previous layer to a probability map of fungal decay. Further information on the network are given in ^6,7^.

Due to the small patch size, the number of pooling layers had been reduced compared to the original U-Net paper ^6^.

We utilized a softmax activation function to allow for easily extending the approach to multi-class segmentation. The network weights were optimized using stochastic gradient descent (SGD) over 200 epochs and a categorical cross-entropy loss function. For regularization, we added several dropout layers ^8^ with a rate of 20% between the convolutional layers in each stage of the network to be robust against over-fitting. The network was trained with 70,000 randomly extracted patches from the training dataset. Thus, training utilized 60% of the available data set and validation and test splits 20% each. Learning rate was set to 0.01. The network weights of the epoch with the best validation loss were used for evaluation on the test dataset.

**Longitudinal view**


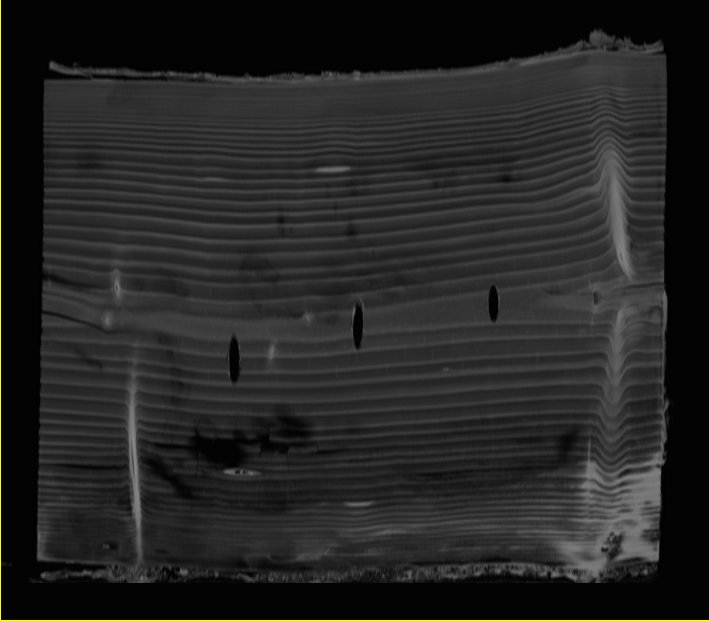

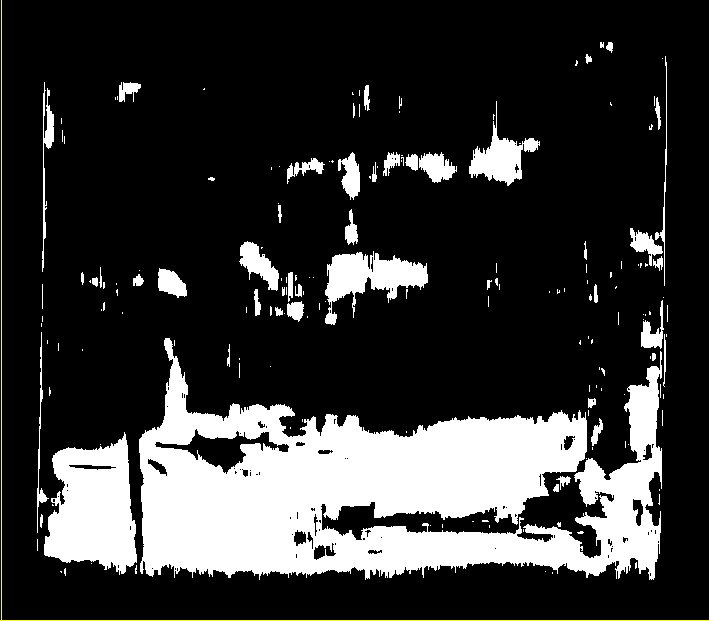

**Figure S2:** Exemplary longitudinal view along the axis of the log generated by combining 709 slices. Left image represents the original CT recording (with device bed removed), right image the binary output of the CNN with with white pixels indicate regions affected by fungi.

**Quality indicators**

F1 = TP/(TP + 0.5 (FP + FN))
ACC = (TP + TN)/(P+N)

SEN = TP/P
SPC = TN/N
TP/PP

P: actal positive (all cases in fact affected by fungi)
N: actual negative (all cases in fact not affected by fungi)
PP: predicted positive (all cases predicted as affected by fungi)
TP: true positive (cases predicted as mold that in fact are affected by fungi)
FP: false positive (cases predicted as mold but in fact are not affected by fungi)

**References**

1. Ehnström, B. & Axelsson, R. *Insektsgnag i bark och ved*. (ArtDatabanken SLU, 2002).

2. Möller, G. Struktur- und Substratbindung holzbewohnender Insekten, Schwerpunkt Coleoptera - Käfer. Dissertation at Freien Universität Berlin. (Freie Universität Berlin, 2009).

3. Seibold, S. *et al.* Association of extinction risk of saproxylic beetles with ecological degradation of forests in Europe. *Conserv. Biol.* **29**, 382–390 (2015).

4. Steger, C., Ulrich, M. & Wiedemann, C. *Machine Vision Algorithms and Applications*. (Wiley, 2008).

5. De Ligne, L. *et al.* Studying the spatio-temporal dynamics of wood decay with X-ray CT scanning. *Holzforschung* **76**, 408–420 (2022).

6. Ronneberger, O., Fischer, P. & Brox, T. *U-net: Convolutional networks for biomedical image segmentation.* (Springer, 2015).

7. Liskowski, P. & Krawiec, K. Segmenting Retinal Blood Vessels with Deep Neural Networks. *IEEE Trans. Med. Imaging* **35**, 2369–2380 (2016).

8. Srivastava, N., Hinton, G., Krizhevsky, A., I. & Salakhutdinov, R. Drop-out: a simple way to prevent neural networks from overfitting. *J. Mach. Learn. Res.* **15**, 1929–1958 (2014).
